# Electrochemical Light-Emitting Gel

**DOI:** 10.3390/ma3063729

**Published:** 2010-06-19

**Authors:** Nobuyuki Itoh

**Affiliations:** Research and Development Center, Dai Nippon Printing Co., Ltd. / 250-1 Wakashiba, Kashiwa, Chiba, 277-0871, Japan; E-Mail: Itou-N9@mail.dnp.co.jp; Tel.: +81-471-341-004; Fax: +81-471-332-540.

**Keywords:** gel, luminescence, electrochemical, ionic liquid, nanoparticle

## Abstract

Light-emitting gel, a gel state electroluminescence material, is reported. It is composed of a ruthenium complex as the emitter, an ionic liquid as the electrolyte, and oxide nanoparticles as the gelation filler. Emitted light was produced via electrogenerated chemiluminescence. The light-emitting gel operated at low voltage when an alternating current was passed through it, regardless of its structure, which is quite thick. The luminescence property of the gel is strongly affected by nanoparticle materials. TiO_2_ nanoparticles were a better gelation filler than silica or ZnO was, with respect to luminescence stability, thus indicating a catalytic effect. It is demonstrated that the light-emitting gel device, with quite a simple fabrication process, flashes with the application of voltage.

## 1. Introduction

Electrogenerated chemiluminescence (ECL) is a well-known luminescence phenomenon of organic molecules that is based on oxidation reduction [1]. Many studies have been conducted on the use of ECL for light sources and display devices [2]. Upon applied voltage, electrons are transferred from the electrodes,through the electric double layer, to the ECL substance. It is different from organic light emitting diodes (OLEDs). There is no requirement for using low work function metals, such as Ca or Li, as a cathode, which are awfully reactive with oxygen and moisture. Both solid and liquid states ECL devices were reported. Solid state ECL devices consist of luminescent chromophore thin films a few submicrometers thick that are between electrodes with a solid electrolyte, such as ionic metal complex, or conductive polymer [1,3]. They have the practical issue of a long light emitting response time against an applied voltage because of the slow ion mobility in the solid film. Liquid state ECL devices usually consist of an electrolyte solution in which the luminescent material and the supporting salts for the organic solvent are dissolved and set between electrodes [1,4]. There are no problems of response time for liquid state ECL devices because of fast diffusion of ions in solution. Liquid state ECL devices can be operated not only by a direct current, but also by an alternating current [5]. The cell thickness, or the gap between electrodes in an AC driven ECL is more than several micrometers, and fabrication of the apparatus is easier than that of other thin film devices. This is quite wide compared to OLEDs, which suffer from problems related to electrical short circuits due to the thin film organic layer of only a few hundred nanometers [6]. By using a nanohole array structure on the electrode similar to the dye-sensitized solar cells [7], the luminescence efficiency improvement was also reported [8].

The author reported a gel state ECL material, the light-emitting gel that operates on low voltage, which prevents the problem of solvent leakage and volatility at the same time [9]. The peak voltage, Vpp, was less than 8 V at 60 Hz, regardless of the considerably thick film structure of more than 100 μm. The light-emitting gel was composed of a ruthenium complex as the emitter, an ionic liquid as the electrolyte, and fumed silica nanoparticles as the gelation filler. Because the vapor pressure was almost zero, the ionic liquid was involatile. The gel was easy to form into various shapes and to print, and emitted light even when it was printed onto a conventional fabric. Although it was expected that the light-emitting gel was suitable for printable electroluminescence material, the brightness and stability are not yet sufficient and the mechanism of annihilation is not understood well enough.

Here, an enhancement of brightness and stability of the light-emitting gel is reported by investigating the electrochemical stability of the ionic liquid and the electrode. In addition, nanoparticles of catalytic materials, such as TiO_2_ and CeO_2_, were also investigated as a gelation filler.

## 2. Results and Discussion

**Figure 1:**
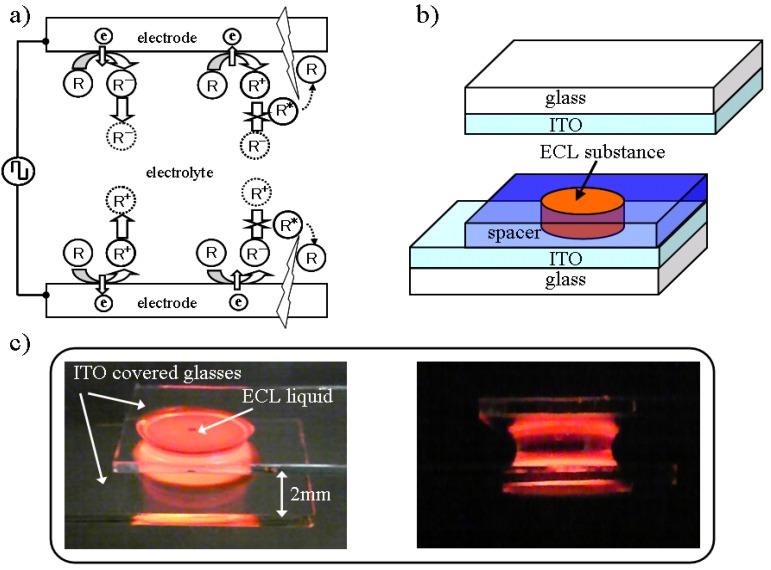
(a) Illustration of AC driven ECL mechanism. (b) Schematic illustration of ECL cell structure and fabrication. (c) Visible appearance of emission between ITOs from ECL liquid. Cross section of emitting ECL liquid (right).

Figure 1a shows the basic mechanism of the AC driven ECL. Where R is an organic molecule, R^−^, R^+^, and R^*^ represent an anion, a cation, and an excitation molecule, respectively. The electrochemical reaction is sequentially processed near the electrode surfaces. In the near side of one electrode surface, an anion is generated in one half period and diffused; a cation is generated and diffused in the next half period. An anion is still drifting near the electrode at this time. The opposite redox reaction is generated near the other side of the electrode surface and, as a result, the anion and cation collide with each other close to both sides of the cell independently, thereby enabling a wide cell gap.

The ECL device structure and fabrication were quite simple, as shown in Figure 1b. The ECL substance, as a liquid or gel, was placed between glass substrates that have transparent electrodes, such as an indium tin oxide (ITO), by controlling the gap with a film spacer of a certain thickness that has a circular opening area. Figure 1c shows how the ECL liquid emits light when low voltage is applied as a square wave of 8 Vpp at 60 Hz, regardless of the thick structure and the very wide electrode gap of 2 mm. The ECL liquid that contains 1 mol % of ruthenium complex Ru(bpy)_3_(PF_6_)_2_ to an ionic liquid, ABImTFSI, was held between ITO glass substrates via surface tension. It is visibly understood that only the electrode neighborhood area emitted light. In the case of ionic Ru(bpy)_3_(PF_6_)_2_, the cation is Ru^3+^ and the anion is Ru^1+^.

**Figure 2:**
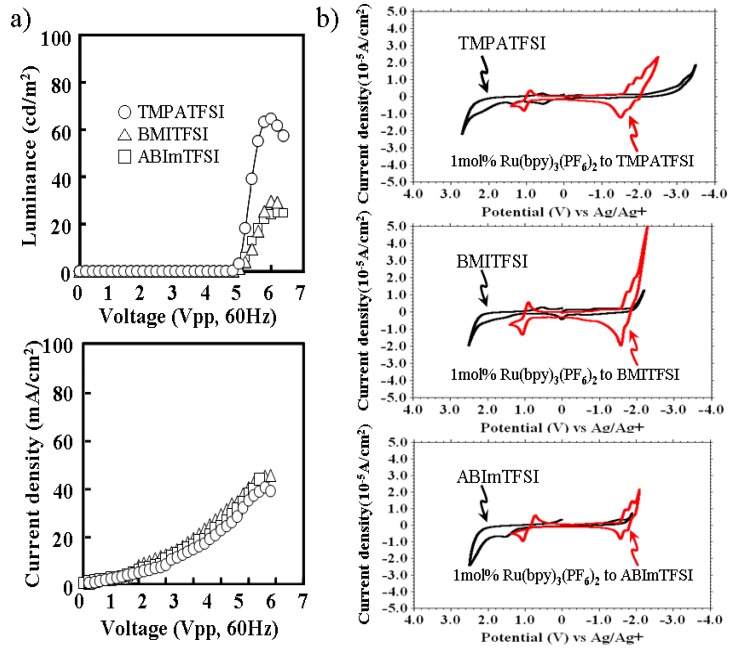
(a) Characteristic of luminance and current density of voltage applied to ECL liquid for different ionic liquids. (b) Cyclic voltammograms of ECL liquid and ionic liquid for each ionic liquid.

Characteristics of ECL liquids, which contain different ionic liquids, are shown in Figure 2. The luminance and current density of the voltage applied are shown in Figure 2a. Figure 2b shows the cyclic voltammograms of each ECL liquid in comparison with each ionic liquid. Each ECL liquid contains 1 mol % of Ru(bpy)_3_(PF_6_)_2_ to each ionic liquid in which the cations were TMPA, BMI, and ABIm. The anion was TFSI, and was the same for all. TMPATFSI showed higher luminance than other ionic liquids, regardless of the same current density. It was found that the wide potential window of ionic liquid, especially for reduction, was preferable in preventing the faraday current branch to the ionic liquid.

The turn-on voltage of 5 Vpp in Figure 2a seems to be high with respect to what is normally obtained with Ru complexes. In the data of CV in Figure 2b, a redox potential difference of the Ru complex is 3 V, and is given by +1 V and −2 V. When the symmetrical square wave, without any offset voltage, was examined to the light emitting device, it was necessary to apply ±2 V in order to give a reductive potential of −2 V. In addition, the conductivity of the transparent ITO used for the actual light emitting device was much lower than that of the platinum electrodes used for the CV experiment. The electric conductivity of ITO was about 0.5 × 10^6 ^(S/m), while it was about 10 × 10^6 ^(S/m) for the platinum. It was thought that the efficient voltage applied to the ECL liquid was slightly up. The turn-on voltage was down, and was 4.5 Vpp, as shown in Figure 4b and Figure 5b when the platinum electrode was used for one side.

**Figure 3:**
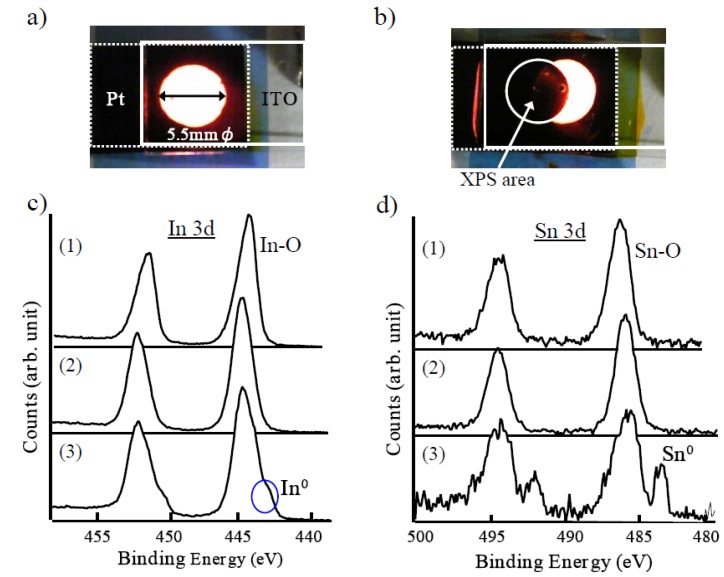
(a) Light emission from the ECL liquid between Pt and ITO. (b) Strange emission pattern when the position of ITO was moved after it was extinct once. See text for explanation of figure. (c) XPS spectrum of ITO In 3d orbital. (1) Intrinsic ITO, (2) Positive DC applied ITO, (3) AC applied ITO and negative DC applied ITO. (d) XPS spectrum of ITO Sn 3d orbital. (1), (2), (3) are the same as (c).

An ECL liquid composed of 1 mol % of Ru(bpy)_3_(PF_6_)_2_ to TMPATFSI was held between ITO and a Pt electrode, as shown in Figure 3a, in order to investigate the electrochemical stability of ITO. When the square wave voltage of 6 Vpp at 60 Hz was applied, the light emission was confirmed to have been disappearing within minutes. Next, the position of ITO was slightly changed by sliding the glass substrate without changing the ECL liquid or the position of Pt. Then, interesting light emission of the crescent shape occurred as shown in Figure 3b. It was found that the short term light emission was primarily dominated by ITO degradation. Three types of ITO sample were prepared in addition to the intrinsic one. An AC applied ITO was prepared by separating the ECL device of Figure 3b. The other two samples were prepared by applying the positive and negative DC of 3 V to the liquid for a certain time with earthed Pt electrode, respectively. Composition elements of each ITO sample were analyzed by the XPS (X-ray photoelectron spectroscopy). Consequently, the measured spectrum for ITO processed by AC and negative DC were almost the same. Figure 3c and 3d show a spectrum of In 3d and Sn 3d orbitals for (1) intrinsic ITO, (2) processed ITO by positive DC, and (3) processed ITO by AC and negative DC, respectively. There was no difference in spectrum between intrinsic ITO and positive DC processed ITO. On the other hand, it was significantly different from the intrinsic ITO for that which was processed by AC and negative DC. This difference is indicative of the metallic elements of In and Sn. These figures indicate that metallic elements, especially Sn, were reduced from ITO by applying a negative component of AC.

Although other transparent electrodes, such as indium zinc oxide (IZO), antimony doped tin oxide (ATO), gallium doped zinc oxide (GZO), and F-doped tin oxide (FTO) were also examined, the issue of the short term emission of light has not been solved yet.

**Figure 4:**
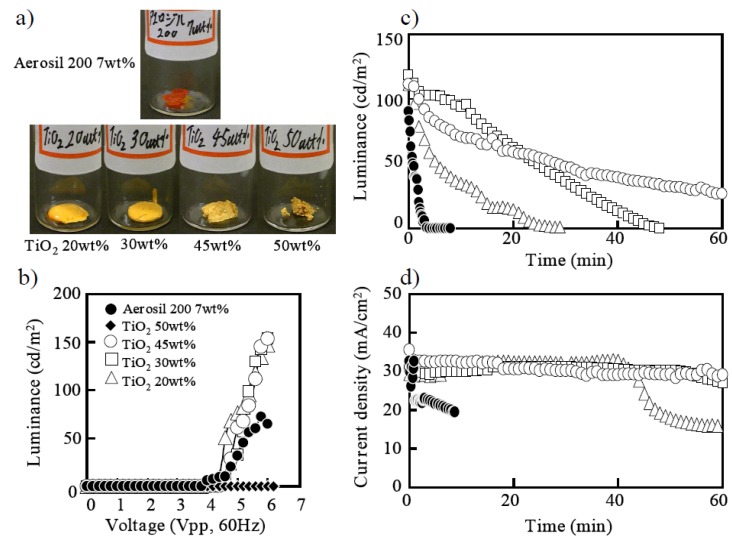
(a) Photographs of ECL gels solidified by silica and TiO_2_ nanoparticles. See text for explanation of figure. (b) Characteristics of luminance of voltage applied to ECL gels. (c) Duration characteristic of luminance of ECL gels. (d) Time characteristic of current density of ECL gels.

It was first reported that the nanoparticles could be utilized for gelation of ionic liquid in order to create a quasi-solid-state electrolyte for dye-sensitized solar cells [10]. It was found that dispersion of fumed silica nanoparticles into ionic liquid induced solidification. Solar cells based on a quasi-solid-state electrolyte exhibited the same efficiency as those with the original ionic liquid. Although the macroscopic viscosity of the gel electrolyte was much higher than the original ionic liquid, equivalent voltammograms were obtained, indicating that the silica nanoparticle network did not disturb ion transfer. Initially in this study, fumed silica nanoparticles were also used for gelation of ELC liquid [9]. An upper photograph of Figure 4a shows an ECL gel obtained by adding 7 wt % of fumed silica nanoparticles (with 12 nm primary particle size) to the ECL liquid and kneading them together. The silica ECL gel emitted light when AC voltage was applied, as shown in Figure 4b; when it was placed between electrodes of ITO and Pt, which is the same as for the ECL liquid. It seemed that the ion diffusion in viscous ionic gel was not disturbed, even under the fast AC electric field, which is not the case in regard to slow natural diffusion of solar cells. As shown in Figure 4c, with the current attenuation in Figure 4d, it became dark in a few minutes and the problem of short luminescence duration was not solved.

Next the author focused on the electrochemically active TiO_2_ nanoparticles, instead of just silica, as a dielectric. Appearances of ECL gel with various amounts of TiO_2_ nanoparticles are shown in the bottom photographs of Figure 4a. The primary size of TiO_2_ nanoparticles were 36 nm. For 20 wt % of TiO_2_ nanoparticles, the liquidity of ionic liquid remained and it was not gelled sufficiently. On the other hand, the softness of gel was lost at 50 wt % of TiO_2_ nanoparticles, and it began to collapse. The range of 30 wt % to 45 wt % of TiO_2_ nanoparticles were good for gelation and easy to handle.

The characteristics of the luminance of each gel with different TiO_2_ densities are shown in Figure 4b, in comparison with the case with the silica nanoparticle. TiO_2_ ECL gel showed much higher brightness without increase in voltage than that of silica ECL gel. It was considered that the harder composite with 50 wt % of TiO_2_ nanoparticles did not emit light because of the prohibiting ionic diffusion. Figure 4c shows the duration characteristics of the light emission that starts from the luminance of 100 (cd/m^2^), and the time characteristic of current density is shown in Figure 4d. It was found that the duration characteristic of the light emission was improved more greatly than silica as increasing the TiO_2_ nanoparticles with the improvement of the electric current drop, as shown in Figure 4d. The TiO_2_ nanoparticle is known as a semiconductor, and at the same time, a catalyst.

**Figure 5:**
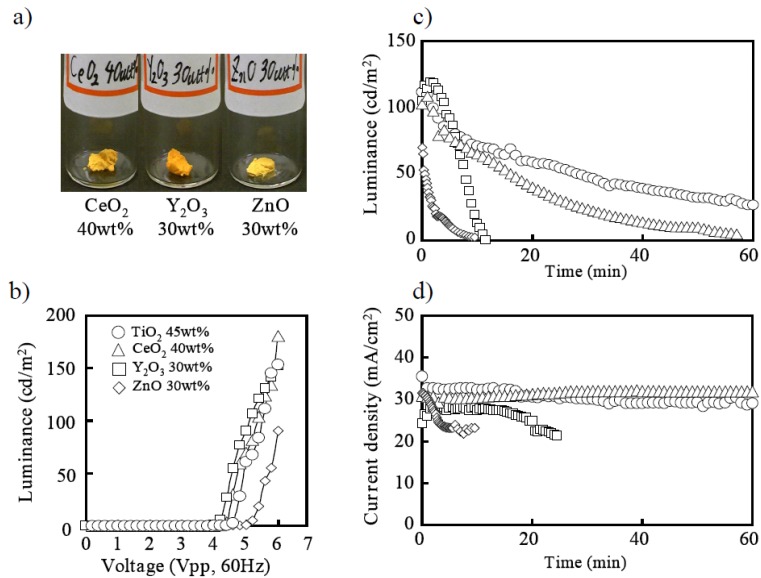
(a) Photographs of ECL gels solidified by CeO_2_, Y_2_O_3_, and ZnO nanoparticles. See text for explanation of figure. (b) Characteristics of luminance of the voltage applied to ECL gels. (c) Duration characteristic of luminance of ECL gels. (d) Time characteristic of current density of ECL gels.

Other semiconductive or catalytic nanoparticles, such as CeO_2_, Y_2_O_3_, and ZnO were examined as gelation filler in comparison with TiO_2_. Photographs of the optimized gels are shown in Figure 5a with each composition of each nanoparticle indicated. For every characteristic of the luminance (Figure 5b), the light emission duration (Figure 5c) and the electric current drop (Figure 5d). TiO_2_ was often followed by CeO2, and ZnO was poor in the performance equivalent to silica. It was found that the light emission duration became longer with an increase in the valence number of ionic crystal metal cations, which were Ti^4+^, Ce^4+^, Y^3+^, and Zn^2+^. SiO_2_ was the covalent crystal, which showed no effect in characteristics improvement.

**Figure 6:**
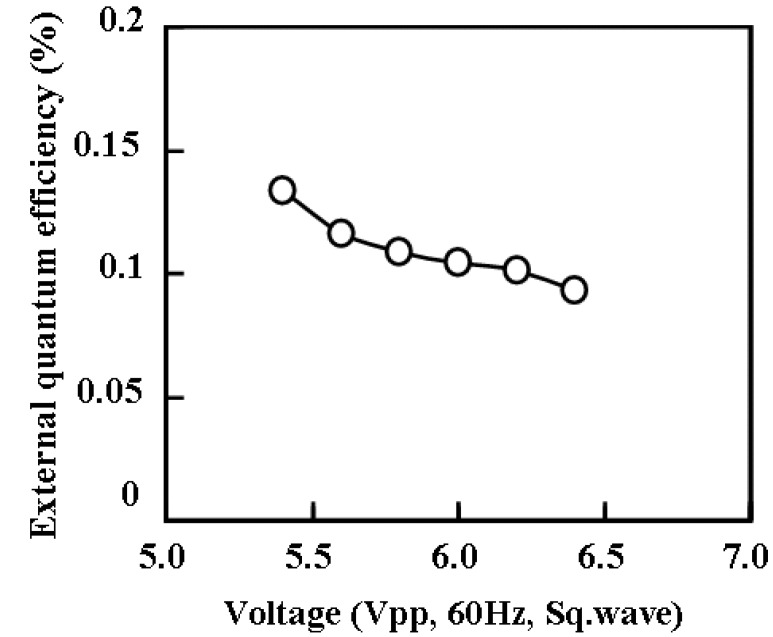
The external quantum efficiency of ECL gel with 45 wt % of TiO_2_ nanoparticles as a function of the applied voltage.

**Figure 7:**
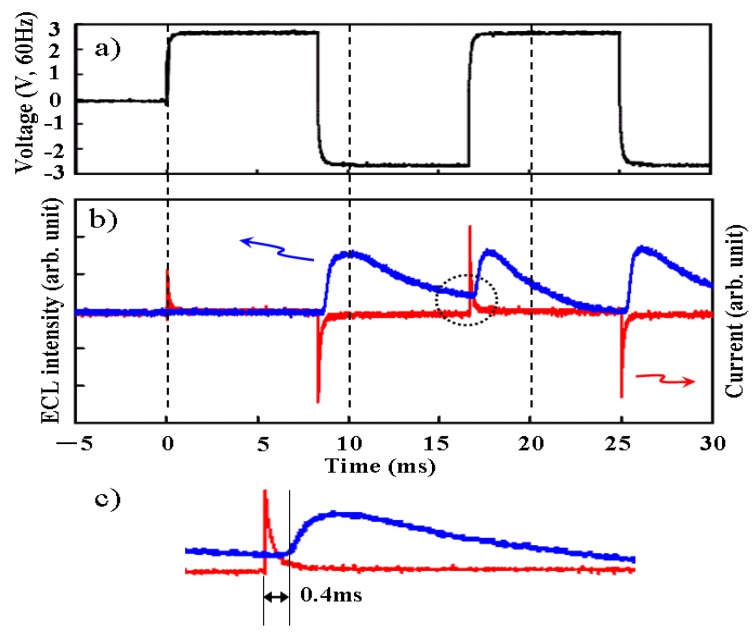
The transient response. (a) Voltage. (b) ECL intensity and current. (c) A magnification of the region enclosed in the dotted line in (b).

**Figure 8:**
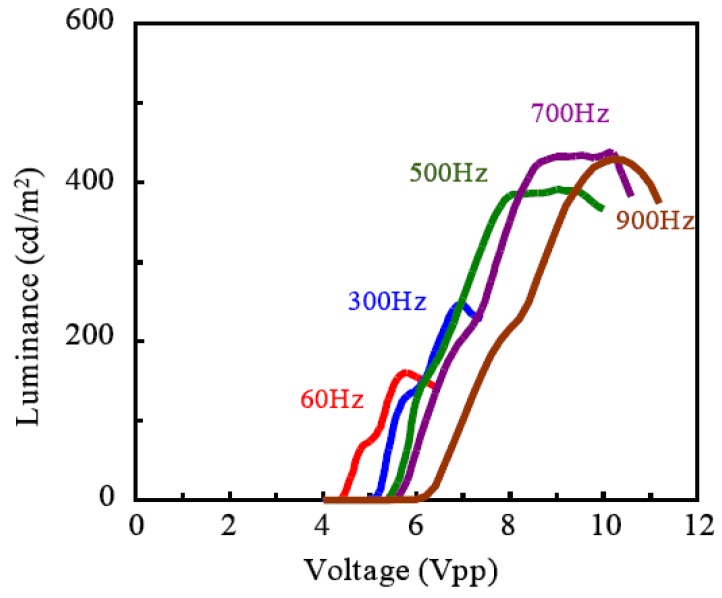
Frequency dependence of luminance characteristic to the voltage applied.

The external quantum efficiency of ECL gel was measured for 45 wt % of TiO_2_ nanoparticles, and shown in Figure 6 as a function of the applied voltage. The transient response of voltage, electric current, and optical intensity were measured simultaneously as shown in Figure 7. It was found that the measured current was mostly non faradic charging the electric double layer. And the light emission began quickly—within 0.4 ms after the charging the electric double layer. Although the apparent efficiency is about 0.1% and lower, the reactive power is just shuttled between the power source and load, and not converted to heat. In fact, temperature rise was not recognized when the light was emitted from ECL gel. The luminance became higher as the frequency was increased, indicating an expansion of withstand voltage, and it reached 400 (cd/m^2^) at 700 Hz as shown in Figure 8.

It is shown in Figure 7 that electric charges are transferred to the Ru complex through the electric double layer in the ELC gel. In other words, it is a luminescence capacitor. The electric double layer is formed with the distortion of the band structure of the electrolyte membrane in the ECL solid, and it is formed by arranging the polarity of the ionic liquid in ECL liquid. It is assumed that the diffused electric double layer around nanoparticles behaves as some electric charge transfer path in the ECL gel, and the emission site may be generated beside the surface of the ITO, which is deoxidized. Thus, the luminescence of ECL gel might be more stable than that of ECL liquid. In the research on the surface potential of the metal oxide particle for the colloidal solutions, it was reported that the zeta potential and iep (isoelectric point) strongly depended on the valence of metal ions. It was suggested that the thickness and electric charge inclination of the diffused electric double layer is influenced. A large difference was reported between trivalent Y_2_O_3_ and tetravalent CeO_2_, similar to observed for ECL gel [12].

When the light-emitting device was assembled, ECL gel was made to stick to the electrode strongly by pressing the glass plate, and the light emission was obtained clearly. This is shown in the video as electronic supplementary information. Because the ionic liquid doesn't evaporate in the vacuum, ECL gel can be observed by using SEM [13] and it will bring us important information.

We are continuing our research, related to the electrokinetic and morphology, on the detailed mechanism of the ECL gel, especially for the TiO_2_ nanoparticle.

## 3. Experimental Section

All the experiments that were sample preparations and electrochemical and optical measurements were performed in open air, not in the nitrogen glove box.

The structure of the ELC device used in the experiment is shown in Figure 1b. The volume of the ECL substance was of a thickness of 80 μm and the diameter of 5.5 mm. A tris(bipyridyl) ruthenium(II) complex, Ru(bpy)_3_(PF_6_)_2_ was prepared by ion exchange following protocols in a previous paper [11]. Ionic liquid materials ABImTFSI, BMITFSI, and TMPATFSI were all from the Kanto Chemical Co., Inc., in which ABIm was 1-allyl-3-butylimidazolium, BMI was 1-butyl-3-methylimidazolium, TMPA was N, N, N-trimethyl-N-propylammonium, and TFSI was bis(trifluoromethanesulfonyl)imide. Viscosities and conductivities of both at 25 °C, were 46 cP and 1.53 mS/cm for ABImTFSI, 52 cP and 4.80 mS/cm for BMITFSI, 72 cP and 3.20 mS/cm for TMPATFSI. ECL liquids were obtained by dissolving 1 mol % of Ru(bpy)_3_(PF_6_)_2_ at 60 °C in each ionic liquid by using a magnetic stirrer for 3 h. An indium tin oxide (ITO) was patterned on a glass substrate by photolithography and HBr etching. Then, ultrasonic washing was done with a neutral detergent and deionized water. Next, it was dried on a hot plate, and finally UV irradiation was conducted.

Cyclic voltammetry measurements were performed under the conditions of the working electrode and the counter electrode of Pt, the reference electrode of Ag/Ag^+^, and the scan rate of 100 mV/s.

The XPS instrument was Theta-Probe, and the X-ray source was a monochromated A1 Kα of 100 W.

Nanoparticles of TiO_2_, CeO_2_, Y_2_O_3_, and ZnO were purchased from CIK NanoTek Co., and the fumed silica nanoparticle Aerosil 200 was purchased from NIPPON AEROSIL Co., the Japanese subsidiary of Evonic Degussa. The primary particle size was 36 nm for TiO_2_, 14 nm for CeO_2_, 33 nm for Y_2_O_3_, 34 nm ZnO, and 12 nm for Aerosil 200 in diameter. ECL gels were obtained by adding prescribed amount of each particle to ECL the liquid and kneading them together as shown in Figure 4a and Figure 5a.

As the voltage was controlled by a synthesized function generator (Yokogawa FG120), the luminance and the AC current density were measured by a luminance meter (Konica Minolta CA-100A) and a digital multimeter (Advantest R6450) respectively.

The external quantum efficiency was measured by using an integrating sphere and a photonic multi-channel analyzer (Hamamatsu Photonics C10027).

The transient response was measured by a digital oscilloscope (Tektronix DPO3014). The electric current was transformed to voltage by inserting a 5 Ω resister in the circuit. The optical intensity was measured by a photo sensor module (Hamamatsu Photonics H7422).

## 4. Conclusions

In summary, the electrochemical light-emitting gel made by using an ionic liquid as the electrolyte and nanoparticles as the solidification filler was presented. The electrochemical light-emitting gel operated with low AC voltages even in a thick structure near 100 μm and in atmosphere. TiO_2_ nanoparticles were a good gelation filler to stabilize the light emission duration. The light-emitting gel has the unique advantage of easy manufacturing. The author envisages that this unique light-emitting material can become lighting technology of high effectiveness and the work will be extended to advanced studies in the future, namely optimization of the device stability.

## Supplementary Files

Supplementary File 1
